# Effects of the factor Xa inhibitor rivaroxaban on the differentiation of endothelial progenitor cells

**DOI:** 10.1186/s12872-023-03318-4

**Published:** 2023-06-02

**Authors:** Ryoichi Sohma, Masashi Sakuma, Syotaro Obi, Setsu Nishino, Ken-ichi Inoue, Satoko Kishimoto, Tianyang Lu, Shigeru Toyoda, Teruo Inoue

**Affiliations:** 1 Center for Advanced Medical Science Research, Dokkyo Medical University, 880 Kitakobayashi, Mibu, Tochigi 321-0293 Japan; 2 Department of Cardiovascular Medicine, Dokkyo Medical University, 880 Kitakobayashi, Mibu, Tochigi 321-0293 Japan; 3 Japan Red Cross Society, Nasu Red Cross Hospital, 1081-4 Nakadawara, Tochigi 324-8686 Otawara, Japan; 4 Dokkyo Medical University, 880 Kitakobayashi, Mibu, Tochigi 321-0293 Japan

**Keywords:** Rivaroxaban, Endothelial progenitor cell, Colony-forming assay, Protease-activated receptor-2, Coronary artery disease

## Abstract

**Background:**

We evaluated the efficacy of the factor Xa inhibitor rivaroxaban on the differentiation ability of vascular endothelial progenitor cells (EPCs), which play roles in vascular injury repair and atherogenesis. Antithrombotic treatment in patients with atrial fibrillation undergoing percutaneous coronary intervention (PCI) is challenging, and current guidelines recommend oral anticoagulant monotherapy 1 year or more after PCI. However, biological evidence of the pharmacological effects of anticoagulants is insufficient.

**Methods:**

EPC colony-forming assays were performed using peripheral blood-derived CD34-positive cells from healthy volunteers. Adhesion and tube formation of cultured EPCs were assessed in human umbilical cord-derived CD34-positive cells. Endothelial cell surface markers were assessed using flow cytometry, and Akt and endothelial nitric oxide synthase (eNOS) phosphorylation were examined using western blot analysis of EPCs. Adhesion, tube formation and endothelial cell surface marker expression was observed in EPCs transfected with small interfering RNA (siRNA) against protease-activated receptor (PAR)-2. Finally, EPC behaviors were assessed in patients with atrial fibrillation undergoing PCI in whom warfarin was changed to rivaroxaban.

**Results:**

Rivaroxaban increased the number of large EPC colonies and increased the bioactivities of EPCs, including adhesion and tube formation. Rivaroxaban also increased vascular endothelial growth factor receptor (VEGFR)-1, VEGFR-2, Tie-2, and E-selectin expression as well as Akt and eNOS phosphorylation. PAR-2 knockdown increased the bioactivities of EPCs and endothelial cell surface marker expression. Patients in whom the number of large colonies increased after switching to rivaroxaban showed better vascular repair.

**Conclusions:**

Rivaroxaban increased the differentiation ability of EPCs, leading to potential advantages in the treatment of coronary artery disease.

**Supplementary Information:**

The online version contains supplementary material available at 10.1186/s12872-023-03318-4.

## Introduction

Dual-antiplatelet therapy using P2Y12 inhibitors and aspirin is the gold standard treatment in patients with coronary artery disease undergoing percutaneous coronary intervention (PCI) to prevent coronary events, such as stent thrombosis [1]. When patients undergoing PCI exhibit concomitant atrial fibrillation, anticoagulant therapy in combination with dual-antiplatelet therapy, i.e., triple antithrombotic therapy, is required [2]. The use of anticoagulant agents in addition to dual-antiplatelet therapy results in an significant increase in the risk of bleeding events [3]. Consequently, selection of the most effective antithrombotic treatment for patients with atrial fibrillation undergoing PCI is a challenge requiring careful assessment of the risks of ischemia and bleeding in each patient, particularly in the drug-eluting stent era.

Recently, as an alternatively to warfarin, anticoagulant therapy directly targeting activated coagulation factor X (FXa) has been established as a treatment strategy for atrial fibrillation [4, 5]. Several clinical and basic studies have suggested that FXa has pro-atherogenic effects beyond blood coagulation [6–8]. These nonhematologic actions are predominantly mediated through activation of protease-activated receptors (PARs) [9–12]. In the process of atherogenesis and subsequent progression of atherosclerosis, risk factors induce vascular injury and then repair mechanisms to counteract injury. When the balance between vascular injury and repair leans toward injury, atherosclerosis develops [13]. Additionally, PCI induces mechanical injury to the vessel wall, but is repaired by tissue regeneration. During vascular injury repair, bone marrow-derived stem cells, including endothelial progenitor cells (EPCs), are mobilized to the injured vessel sites. Then, the EPCs differentiate into vascular endothelial cells, leading to endothelial regeneration, i.e., re-endothelialization, which is essential for vascular repair [14–16]. Therefore, strategies to induce the differentiation of EPCs may be promising for post-PCI management as well as prevention of atherosclerosis progression. We hypothesize that direct oral anticoagulants, i.e., FXa inhibitors, may have beneficial effects on the mobilization and differentiation of EPCs and may therefore be a powerful treatment method in patients who have coronary artery disease and atrial fibrillation.

Thus, in this study, we investigated the effects of the FXa inhibitor rivaroxaban on the behaviors of EPCs using EPC colony-forming assays, evaluated the molecular mechanisms of rivaroxaban, and examined the potential of rivaroxaban to induce re-endothelialization and early vascular healing after PCI.

## Methods

### Materials

Rivaroxaban was purchased from ChemScene LLC (Monmouth Junction, NJ, USA), and was dissolved in dimethyl sulfoxide (DMSO) before use. Warfarin sodium was purchased from Wako Pure Chemical Industries (Osaka, Japan).

### Isolation and preparation of human CD34-positive cells

For EPC colony-forming assays, human mononuclear cells were isolated by density gradient centrifugation using Histopaque1077 (Sigma-Aldrich, St. Louis, MO, USA) from heparinized peripheral blood of healthy volunteers and patients with chronic coronary artery disease who underwent coronary stent implantation. The mononuclear cells were also isolated from umbilical cord blood (Riken BRC Cell Bank, Tsukuba, Japan) for other experiments. CD34-positive cells were purified using anti-CD34 monoclonal antibody-conjugated microbeads (Miltenyi Biotec, Bergisch Gladbach, Germany) and a magnetically activated cell sorter (auto MACS; Miltenyi Biotec) following the manufacturer’s protocol. The isolated cells contained approximately 95% pure CD34-positive cells.

### EPC colony-forming assays

Human CD34-positive cells were prepared from the peripheral blood of healthy volunteers as well as coronary artery disease patients. Approximately 2 × 10^3^cells were cultured in methylcellulose (H4236; StemCell Technologies, Vancouver, BC, Canada) with 100 ng/mL stem cell factor (SCF), 50 ng/mL vascular endothelial growth factor (VEGF), 20 ng/mL interleukin (IL)-3, 50 ng/mL basic fibroblast growth factor (bFGF), 50 ng/mL epidermal growth factor (EGF), 50 ng/mL insulin-like growth factor-1 (IGF-1; all from PeproTech, Rocky Hill, NJ, USA), and 2 U/mL heparin (Wako Pure Chemical Industries, Osaka, Japan) in 3.5-cm dishes. After 21 days, colony formation was observed. We counted the number of total colonies and colonies for small- or large-type EPCs in dishes using a phase-contrast microscope (CK30; Olympus, Tokyo, Japan). Small- and large-type EPCs were identified by visual inspection with an inverted microscope under 40 × magnification. Small-type EPCs were composed of round adhesive cells, and large-type EPCs were composed of spindle-shaped cells [17, 18].

### Ex-vivo suspension culture of EPCs

CD34-positive cells isolated from umbilical cord blood were expanded as previously reported [17]. Briefly, 4 × 10^5^ cells were cultured at 37 °C in an atmosphere containing 5% CO_2_ in Stem Span medium (StemCell Technologies) with 50 ng/mL VEGF, 20 ng/mL IL-6, 100 ng/mL SCF, 20 ng/mL thrombopoietin (PeproTech), and 100 ng/mL fms-related tyrosine kinase 3 ligand (PeproTech) in 24-well plates for 7 days. Ex-vivo expanded CD34-positive cells (1.5 × 10^6^ cells) were seeded in 10-cm culture dishes coated with human fibronectin (Corning, Bedford, MA, USA) and then cultured in M199 medium (Gibco; Thermo Fisher Scientific, Grand Island, NY, USA) supplemented with 5% fetal bovine serum (Sigma-Aldrich), 10% dextran (molecular weight: 100,000–200,000; cat. no. D4876; Sigma-Aldrich), and endothelial growth medium containing VEGF, bFGF, EGF, IGF-1, and ascorbic acid (EGM-2; Lonza, Basel, Switzerland) at 37 °C in an atmosphere containing 5% CO_2_. After 7 days of culture, adhesive cells were used as EPCs.

### Adhesion assay

EPCs (2 × 10^4^) in suspension with or without rivaroxaban for 7 days were seeded onto human fibronectin-coated 96-well plates in M199 medium with 10% fetal bovine serum (FBS) and 10% dextran. After incubation with 5% CO_2_ for 6 h, nonadherent cells were removed by gently washing three times with phosphate-buffered saline (PBS). Adhesive cells were examined under a phase-contrast microscope equipped with a digital camera. The number of adherent cells per image was measured.

### Tube formation assay

For analysis of tube formation, 2 × 10^3^ EPCs and 1.5 × 10^4^ human umbilical vein endothelial cells (HUVECs) were cultured with or without rivaroxaban in EBM-2 medium (Lonza) with 2% FBS and added to an equivalent amount of Matrigel (BD Falcon; Becton Dickinson, San Jose, CA, USA) in 96-well plates. After incubation at 37 °C in an atmosphere containing 5% CO_2_, gels were observed using phase-contrast microscopy. The number of circles per tube structure was counted in each image.

### Flow cytometry

EPCs were washed with cold PBS and resuspended in PBS with FcR blocking reagent (Miltenyi Biotec), 2% FBS, and 2 mM ethylenediaminetetraacetic acid (EDTA) at 4 °C for 15 min. Cells were then stained with phycoerythrin (PE)-conjugated monoclonal antibodies specific for the following surface antigens: CD31 (Becton Dickinson), VEGF receptor (VEGFR)-1 (Becton Dickinson), VEGFR-2 (Becton Dickinson), protein receptor tyrosine kinase, epithelial-specific (Tie)-2 (R&D Systems, Minneapolis, MN, USA), and E-selectin (CD62E; Becton Dickinson). After incubation at 4 °C for 30 min, cells were washed twice with PBS and analyzed using a FACS Calibur flow cytometer (Becton Dickinson).

### Western blot analysis

Cell lysates were prepared using ice-cold lysis buffer (50 mM Tris–HCl, 150 mM NaCl, 1 mM EDTA, 1 mM ethylene glycol tetraacetic acid, 1% NP-40, 10% glycerol, 20 mM Na_4_P_2_O_7_·10H_2_O, 200 mM NaF, and 1 mM Na_3_VO_4_ plus phosphatase and protease inhibitor). Samples (20–35 μg total protein for Akt/endothelial nitric oxide synthase [eNOS] experiments and 200 μg total protein for PAR-2 knockdown experiments) were separated by sodium dodecyl sulfate polyacrylamide gel electrophoresis using 6% or 8% gels and then transferred to Amersham Hybond P PVDF 0.45 membranes (GE Healthcare UK, Buckinghamshire, UK). The blots were blocked with Blocking One (Nacalai Tesque, Kyoto, Japan) for 1 h at room temperature, incubated overnight at 4 °C with primary antibodies, and washed three times with Tris-buffered saline/0.1% Tween 20 (TBS-T). Membranes were then incubated with horseradish peroxidase-conjugated anti-rabbit (anti-mouse for glyceraldehyde-3-phosphate dehydrogenase [GAPDH]) IgG antibodies, according to standard methods. Immunoreactive signals were enhanced with Chemi-Lumi One Super (Nacalai Tesque) or Chemi-Lumi One Ultra (Nacalai Tesque) and visualized using a WSE-6100H LuminoGraph I (ATTO, Tokyo, Japan). The intensity of each band was measured using a CS Analyzer 4 (ATTO). Quantitative analysis was achieved by normalization of the signal of each protein to that of GAPDH or β-actin. Anti-eNOS, anti-phospho-eNOS (Ser1177), anti-Akt, anti-phospho-Akt (Ser473), anti-β-actin, and anti-PAR-2 antibodies were obtained from Cell Signaling Technology (Danvers, MA, USA). Anti-GAPDH antibodies were obtained from Novus Biologicals (Centennial, CO, USA).

### Transfection with synthetic small interfering RNA (siRNA)

PAR-2 siRNA and control siRNA (scrambled siRNA) were purchased from Invitrogen (Carlsbad, CA, USA). PAR-2 siRNA and control siRNA were transfected into EPCs to a final concentration of 5 nM with 0.3% Lipofectamine RNAiMax (Invitrogen). Transfected cells were incubated at 37 °C in an atmosphere containing 5% CO_2_ for 7 days before use in experiments.

### Clinical experiments

In the clinical investigation, patients with chronic coronary artery disease (i.e., stable effort angina or old myocardial infarction) and atrial fibrillation, who underwent PCI using drug-eluting stents, were enrolled. These patients were receiving an anticoagulant warfarin in addition to single antiplatelet therapy with aspirin (100 mg/day) until undergoing PCI. Blood coagulation activity was adequately suppressed by warfarin treatment as the 2.0–3.0 international normalized ratio of the prothrombin time. Immediately after PCI, the patient received dual antiplatelet therapy by adding a P2Y12 inhibitor prasugrel (loading dose: 20 mg, maintenance dose: 3.75 mg/day) to aspirin. Simultaneously the patients switched anticoagulant therapy from warfarin to rivaroxaban. For rivaroxaban prescription, the dosage was selected based on recommendations in Japan (10 mg/day for patients with a creatinine clearance of 15–49 mL/min or 15 mg/day for patients with a creatinine clearance of ≥ 50 mL/min). In these patients, we observed changes in circulating EPCs during the acute phase after PCI and assessed stented-site vascular healing during the chronic phase.

We collected peripheral blood to observe the circulation EPCs before PCI under the warfarin prescription and 7 days after PCI under the rivaroxaban prescription. First, we measured the number of cells positive for both CD34 and kinase insert domain receptor (KDR) (CD34^+^/KDR^+^cells) as cell fractions including abundant EPCs using flow cytometry as previously described [19]. We then performed qualitative assessment using EPC colony-formation assays.

At follow-up coronary angiography 12 months after PCI, we assessed stented-site vascular healing by observing neointimal coverage over the stent strut using optical coherence tomography (OCT), as previously reported [19, 20]. Briefly, cross-sectional OCT images were analyzed at 0.6-mm intervals. In cross-sectional images, neointimal coverage was assessed for all struts, including uncovered struts and malapposed struts. The definition of malapposed struts was described by Sakuma et al. [19] The percentage of uncovered struts to total struts and that of malapposed struts to total struts was then calculated for all OCT cross-sections.

### Statistical analysis

**Fig. 1:**
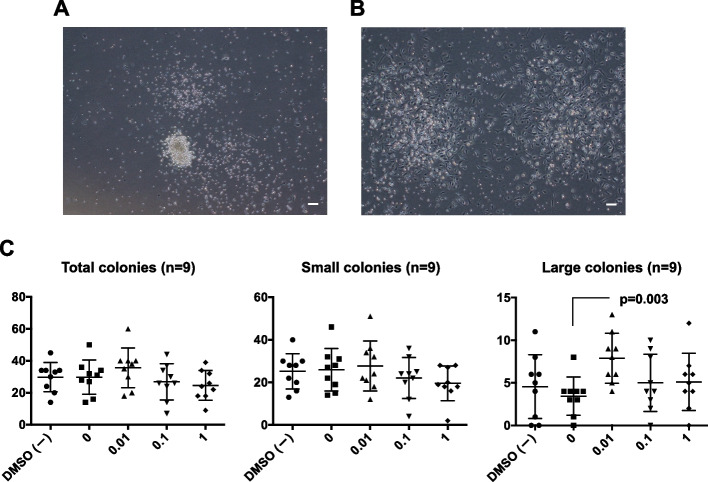
EPC colony-forming assays. **A** Colonies of primitive, dense, small, round EPCs showed higher proliferation potential and were composed of immature EPCs. Scale bar: 100 μm. **B** Colonies of definitive, large, spindle-type EPCs showed more vasculogenic properties and were composed of differentiating EPCs. Scale bar: 100 μm. **C** Effects of rivaroxaban on the numbers of total EPC colonies, small EPC colonies, and large EPC colonies. EPC: endothelial progenitor cell; DMSO, dimethyl sulfoxide. Bars are means ± standard deviations of nine samples

**Fig. 2:**
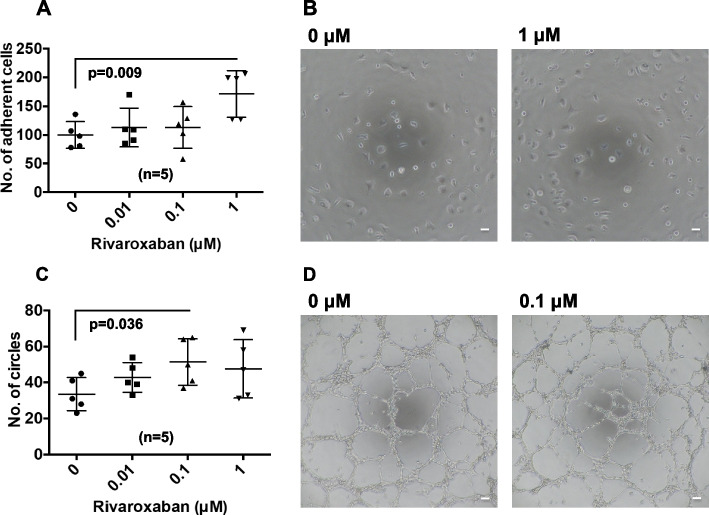
Effects of rivaroxaban on adhesion and tube formation. **A** EPCs exposed to 0.1% DMSO (control) or rivaroxaban for 7 days were cultured for 6 h. The number of adhesive cells per low power field was counted. Bars are means ± standard deviations of five samples. **B** The adhesive cells were observed by phase-contrast microscopy. Scale bar: 100 μm. **C** EPCs exposed to 0.1% DMSO (control) or rivaroxaban for 7 days were cultured in Matrigel with human umbilical vein endothelial cells. The number of tubes per low power field was measured after 5 h. Bars are means ± standard deviations of five samples. **D** The tube formation observed by phase-contrast microscopy. Scale bar: 100 μm

**Fig. 3:**
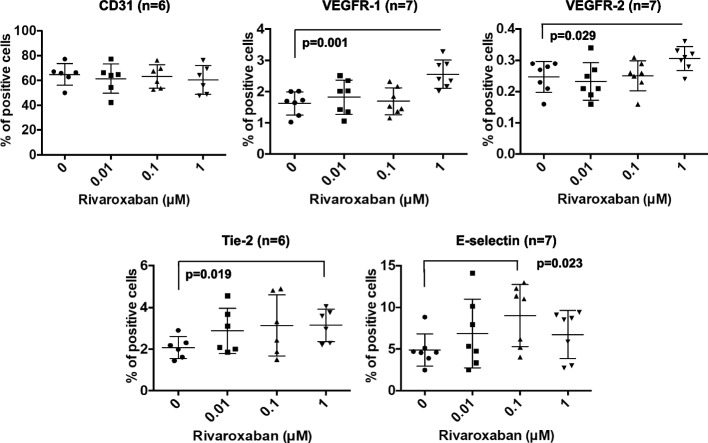
Flow cytometric analysis of the expression of endothelial cell markers on the surface of umbilical cord blood-derived CD34-positive cells. The effects of DMSO alone (control) and rivaroxaban on the percentages of cells positive for VEGFR-1, VEGFR-2, Tie-2, E-selectin, and CD31 were assessed. VEGFR: vascular endothelial growth factor receptor. Bars are means ± standard deviations of six or seven samples

**Fig. 4:**
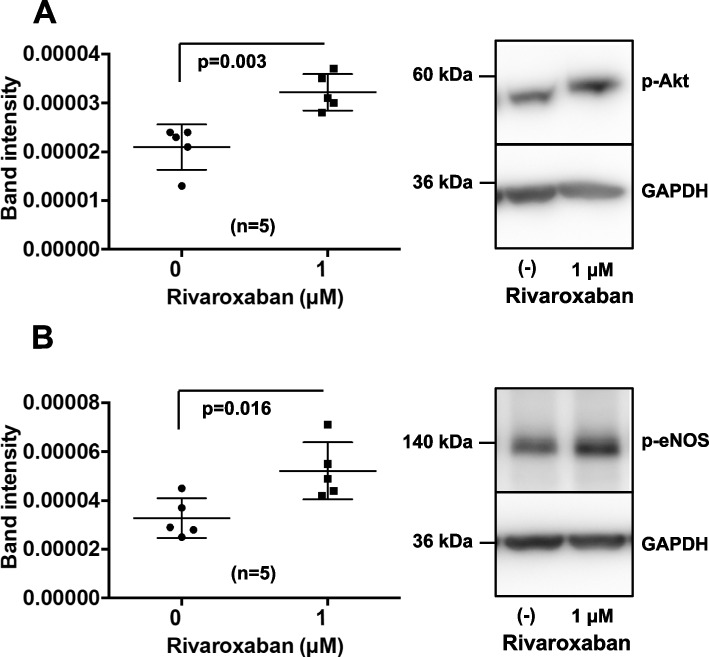
Western blot analysis of Akt/eNOS phosphorylation. **A** The effects of DMSO (control) and rivaroxaban on Akt phosphorylation levels were evaluated. p-Akt: phosphorylated Akt; GAPDH, glyceraldehyde-3-phosphate dehydrogenase. Bars are means ± standard deviations of five samples. **B** The effects of DMSO (control) and rivaroxaban on eNOS phosphorylation levels were evaluated. p-eNOS: phosphorylated endothelial nitric oxide synthase. Bars are means ± standard deviations of 5 samples. The original images without crop and correction are shown in the Additional file 2: Figure S2

**Fig. 5:**
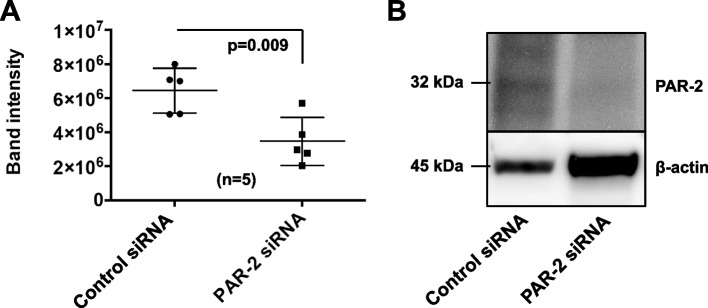
Transfection with synthetic PAR-2 siRNA into EPCs. A: The effect of transfection with synthetic PAR-2 siRNA into EPCs were evaluated by westernblotting. Values are means ± standard deviations of five samples. B: Expression of PAR-2 in EPCs transfected with PAR-2 siRNA compared with that in cells transfected with control siRNA. PAR, protease-activated receptor; siRNA, small interfering RNA. The full-length gels of PAR-2 protein are shown in the Additional file 3: Figure S3 and Additional file 4: Figure S4

**Fig. 6:**
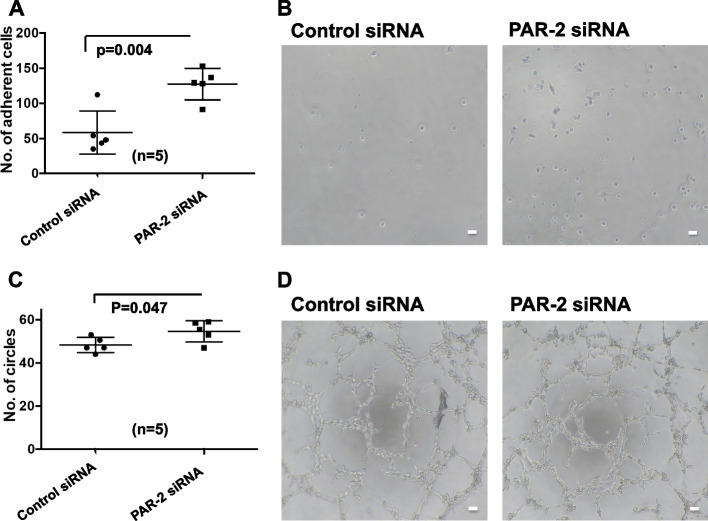
The bioactivities of EPCs transfected with PAR-2 siRNA. **A** EPCs transfected with control or PAR-2 siRNA for 7 days were cultured for 6 h. The number of adhesive cells per low power field was counted. Bars are means ± standard deviations of five samples. **B** The adhesive cells were observed by phase-contrast microscopy. Scale bar: 100 μm. **C** EPCs transfected with control or PAR-2 siRNA for 7 days were cultured in Matrigel with human umbilical vein endothelial cells. The number of tubes per low power field was measured after 5 h. Bars are means ± standard deviations of five samples. **D** The tube formation observed by phase-contrast microscopy. Scale bar: 100 μm

**Fig. 7:**
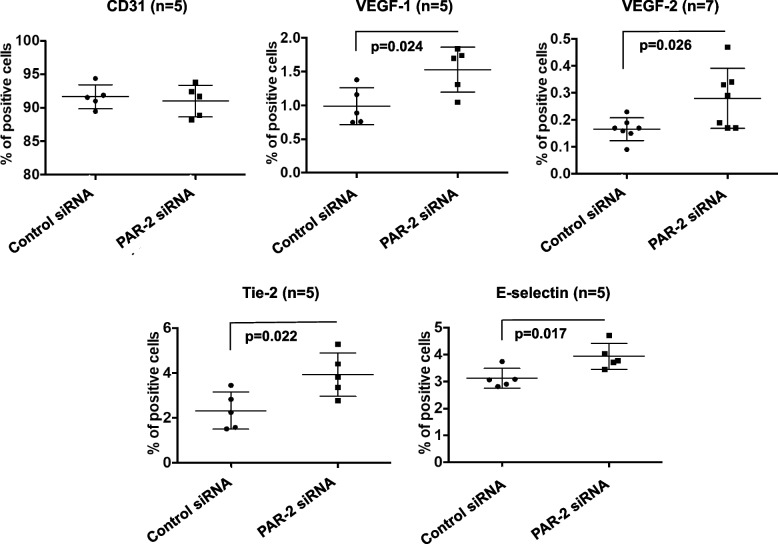
Surface protein expression of endothelial cell markers in PAR-2-deficient. After PAR-2 siRNA transfection, effects of PAR-2 knockdown on surface protein expression of VEGFR-1, VEGFR-2, Tie-2, E-selectin, and CD31 were evaluated. Bars are means ± standard deviations of five samples (seven samples for VEGFR-2)

Values were presented as means ± standard deviations. The effects of serial rivaroxaban concentrations were analyzed using Fisher’s least significant difference procedure as follows: for Fig. 1, Friedman tests (paired nonparametric); for Figs. 2 and 3, analysis of variance followed by Student’s t tests as a post-hoc analysis (parametric); for Figs. 4, 5, 6 and 7, two-group comparisons were performed using Student’s t tests (parametric). Effects on patients were unable to be assessed due to the limited sample size. Results with *P* values less than 0.05 were considered significant.

## Results

### Rivaroxaban enhanced the differentiation ability of human peripheral blood EPCs

To confirm whether rivaroxaban induced the differentiation of EPCs into vascular endothelial cells, we performed EPC colony-forming assays. Observation of the two types of colonies enables prediction of EPC differentiation potential [17, 18]. Colonies composed of primitive, dense, small, round EPCs showed higher proliferation potential and were composed of immature EPCs (Fig. 1A). By contrast, colonies composed of definitive, large, spindle-type EPCs showed more vasculogenic properties and were composed of differentiating EPCs (Fig. 1B). EPC colony-forming assays were performed using CD34-positive cells isolated from 9 healthy humans (5 men and 4 women, ages 37.0 ± 10.2 yrs) to determine whether rivaroxaban affected EPC differentiation. At the beginning of cell culture, rivaroxaban was added to the culture medium at 0.01, 0.1, or 1 μM. Cultures without DMSO or with DMSO alone were used as controls. Compared with the DMSO control, the addition of 0.01 μM rivaroxaban significantly increased the number of large EPC colonies (3.4 ± 2.2 to 7.9 ± 2.9, *P* = 0.003: null hypothesis retained by Friedman test), although it did not change total EPC colonies (29.8 ± 10.7 to 35.7 ± 12.5, P = 0.333) and small EPC colonies (26.0 ± 1.0 to 27.8 ± 11.7, *P* = 0.638). The addition of 0.1 and 1 μM rivaroxaban did not change total EPC colonies (0.1 μM: to 26.9 ± 11.3, *P* = 0.586 and 1 μM: to 24.7 ± 9.3, *P* = 0.333), small EPC colonies (0.1 μM: to 22.1 ± 9.6, *P* = 0.412 and 1 μM: to 19.6 ± 8.1, *P* = 0.638) and large EPC colonies (0.1 μM: to 5.0 ± 3.4, *P* = 0.985 and 1 μM: to 5.1 ± 3.4, P = 0.945) (Fig. 1C). These results suggest that low-dose rivaroxaban may induced the differentiation ability of circulating EPCs. To confirm whether the differentiation ability was specific for rivaroxaban but not for warfarin, we performed similar EPC colony-forming assays also using warfarin sodium. Consequently, warfarin stimulation did not show any significant changes in total colonies, small colonies, or large colonies compared to the control (Additional file 1: Fig. S1).

### Rivaroxaban increased adhesion and tube formation

Adhesion assays were performed to investigate whether rivaroxaban affected the adhesion of umbilical cord blood-derived EPCs. Exposure of EPCs to 1 μM rivaroxaban for 7 days significantly increased the adhesion (Fig. 2A and B). Some EPCs exposed to rivaroxaban became elongated. To investigate whether rivaroxaban affected the ability of EPCs to form capillary-like tubes, tube formation assays were performed. EPCs interconnected and formed tightly adherent cords of cells as early as 2 h after seeding. Formation of tube-like structures became more prominent at 4 h, and an extensive tubular network was observed at 5 h. EPCs exposed to 0.1 µM rivaroxaban increased the tube number (Fig. 2C and D). These findings indicated that rivaroxaban increased the bioactivities of EPCs.

### Rivaroxaban increased endothelial cell surface marker expression in EPCs

Umbilical cord blood-derived EPCs were cultured for another 7 days in human fibronectin-coated 10-cm dishes with or without 0.01, 0.1, or 1 μM rivaroxaban. Culture with DMSO alone was used as a control. Cell surface expression of endothelial markers, such as CD31, VEGFR-1, VEGFR-2, Tie-2, and E-selectin, was analyzed by flow cytometry, and the percentages of marker-positive cells among 2 × 10^4^ events for all EPCs were calculated (Fig. 3). Compared with the DMSO alone control, 1 μM rivaroxaban treatment for 7 days significantly increased the percentages of cells positive for VEGFR-1 (1.62% ± 0.4% to 2.56% ± 0.4%, *P* = 0.001), VEGFR-2 (0.25% ± 0.05% to 0.30% ± 0.04%, *P* = 0.029), and Tie-2 (2.07% ± 0.5% to 3.14% ± 0.8%, *P* = 0.019), and 0.1 μM rivaroxaban also significantly increased that of E-selectin (4.88% ± 1.9% to 9.00% ± 3.7%, *P* = 0.023). Rivaroxaban did not change the percentage of CD31-positive cells. These results suggest that rivaroxaban may induce the differentiation of EPCs into vascular endothelial cells.

### Rivaroxaban increased Akt and eNOS phosphorylation

Because the phosphatidylinositol-3 kinase (PI3K)/Akt/eNOS pathway plays pivotal roles in EPC mobilization, migration, and homing [21], we assessed whether rivaroxaban induced Akt and eNOS phosphorylation using western blotting. EPCs from umbilical cord blood-derived CD34-positive cells were cultured for another 7 days in human fibronectin-coated 6-cm dishes with or without 1 μM rivaroxaban. Addition of DMSO alone was used as a control. Consequently, 1 μM rivaroxaban markedly increased Akt and eNOS phosphorylation compared with the control (Fig. 4A and B). These results suggest that the Akt/eNOS pathway may be an important activator of EPC differentiation in response to rivaroxaban.

### PAR-2 deficiency increased the bioactivities and endothelial cell surface marker expression in EPCs

Because the pro-inflammatory and pro-atherogenic effects of FXa are mediated through activation of PARs, particularly PAR-2 [12], we hypothesized that the effects of rivaroxaban on EPC differentiation may be based on inhibition of FXa/PAR-2 binding and that PAR-2 deficiency may induce EPC differentiation. Therefore, we knocked down PAR-2 in umbilical cord blood-derived EPCs. The efficiency of PAR-2 knockdown by PAR-2 siRNA transfection was analyzed using western blotting. PAR-2 protein levels were significantly reduced in PAR-2 siRNA-transfected EPCs compared with control siRNA-transfected EPCs (Fig. 5A and B). Next, we evaluated the bioactivities and surface protein expression of endothelial markers in the EPCs transfected with PAR-2 siRNA or control siRNA. PAR-2 knockdown significantly increased the bioactivities of EPCs both the adhesive cell number (58.5 ± 30.7 to 127.4 ± 22.4, *P* = 0.004) (Fig. 6A and B) and the tube number (48.3 ± 3.5 to 54.6 ± 4.9, *P* = 0.047) (Fig. 6C and D). Additionally, PAR-2 knockdown significantly increased the surface levels of VEGFR-1 (0.99 ± 0.3 to 1.53 ± 0.3, *P* = 0.024), VEGFR-2 (0.16 ± 0.04 to 0.28 ± 0.11, *P* = 0.026), Tie-2 (2.33 ± 0.8 to 3.93 ± 1.0, *P* = 0.022), and E-selectin (3.12 ± 0.4 to 3.93 ± 0.5, *P* = 0.017), but not the level of CD31 (Fig. 7). These findings might support our hypothesis.

### Clinical experiments

We performed clinical experiments in 4 patients (3 men and 1 woman; ages 69.5 ± 5.7 years). Baseline characteristics, changes in profiles of EPCs, and OCT findings for stented-site vascular healing are shown in Table 1. Compared with baseline under the warfarin prescription, circulating CD34^+^/KDR^+^ cells increased on day 7 after PCI under the rivaroxaban prescription in all patients. Regarding EPC colony-forming assays, in cases 1 and 3, the number of large colonies increased on day 7 after PCI under rivaroxaban, compared with baseline under warfarin. In these 2 patients, the percentages of uncovered and malapposed struts were reduced compared with those in the other 2 patients (cases 2 and 4), in whom the number of large colonies was decreased.

**Table 1:** PCI-induced change in EPCs in patients after switching to rivaloxaban

	Case 1	Case 2	Case 3	Case 4
Age; yr	66	68	66	78
Gender; M/F	M	M	M	F
Body mass index; kg/m^2^	25	23	27	25
Basal disease; AP/OMI	AP	AP	OMI	AP
Lesion location; LAD/LCX/RCA	LAD	RCA	LAD	RCA
Risk factor; Yes/no				
Hypertension	Yes	Yes	Yes	No
Diabetes	No	No	No	Yes
Dyslipidemia	Yes	Yes	Yes	Yes
Smoking	Yes	Yes	Yes	No
Medications; Yes/no				
ACE inhibitors/ARBs	Yes	Yes	Yes	Yes
Statins	Yes	Yes	Yes	Yes
Anti-diabetic agents	No	No	No	Yes
Dosage of rivaroxaban; mg/day	10	15	15	15
CD34 + /KDR + cells; /2$\times$10^5^ MNCs				
Before PCI under warfarin	104	2	4	5
7 days after PCI under rivaroxaban	209	18	108	6
Total colony; n				
Before PCI under warfarin	12	12	14	8
7 days after PCI under rivaroxaban	11	2	15	10
Small colony; n				
Before PCI under warfarin	11	6	10	4
7 days after PCI under rivaroxaban	3	1	8	10
Large colony; n				
Before PCI under warfarin	1	6	4	4
7 days after PCI under rivaroxaban	8	1	7	0
Uncovered strut; %	7.85	44.85	13.27	31.78
Malapposed strut; %	7.58	43.92	13.27	29.02

*M* indicates male, *F* female, *AP* angina pectoris, *OMI* old myocardial infarction, *LAD* left anterior descending artery, *RCA* right coronary artery, *ACE* angiotensin converting enzyme, *ARBs* angiotensin receptor blockers, *KDR* kinase insert domain receptor, *MNCs* mononuclear cells, *PCI* percutaneous coronary intervention

## Discussion

In the present study, we demonstrated that rivaroxaban increased the number of large EPC colonies whereas warfarin did not, suggesting that rivaroxaban might specifically induce definitive-type EPC colony-forming cells. In addition, rivaroxaban increased VEGFR-1, VEGFR-2, Tie-2, and E-selectin protein expression on the surface of EPCs and enhanced adhesion and tube formation of the EPCs. Furthermore, rivaroxaban induced Akt and eNOS phosphorylation in the EPCs. These results suggest that rivaroxaban might increase the differentiation of EPCs into vascular endothelial cells via activation of the Akt/eNOS pathway.

Antithrombotic treatment for patients with coronary artery disease and atrial fibrillation undergoing PCI is challenging, particularly in the drug-eluting stent era, because anticoagulant therapy in combination with dual-antiplatelet therapy, i.e., triple antithrombotic therapy, is required. Triple antithrombotic therapy increases the risk of serious bleeding complications, and thus, current guidelines recommend switching the triple antithrombotic therapy to double antithrombotic therapy or further to oral anticoagulant monotherapy as soon as possible [2]. In these guidelines, however, evidence supporting the pharmacological effects of anticoagulants is insufficient.

As an alternative to warfarin, anticoagulant therapy using direct oral anticoagulants, e.g., FXa inhibitors, has been established as a treatment strategy for atrial fibrillation [4, 5]. The FXa inhibitor rivaroxaban prevents cardiovascular events in atherosclerotic cardiovascular diseases, independently of the prevalence of atrial fibrillation [22, 23]. The Atrial Fibrillation and Ischemic Events with Rivaroxaban in Patients with Stable Coronary Artery Disease (AFIRE) trial demonstrated that rivaroxaban monotherapy was noninferior to double antithrombotic therapy (rivaroxaban and a single antiplatelet drug, i.e., aspirin or a P2Y12 inhibitor) with regard to efficacy and superior with regard to safety in patients with coronary artery disease and atrial fibrillation, even 1 year or more after PCI using drug-eluting stents [24]. The results of AFIRE suggested that rivaroxaban alone prevented ischemic events in the long term and may have antiatherogenic effects. Because the enhanced differentiation ability of EPCs into vascular endothelial cells could explain the anti-atherogenic properties of rivaroxaban, we believe that our findings support the results of the AFFIRE trial.

FXa has various pro-atherogenic activities beyond blood coagulation via the activation of PARs [6–12]. FXa is a potent platelet agonist, and its effects on platelet activation are mediated through PAR-1 and PAR-4 activation. Therefore, rivaroxaban inhibits platelet activation, possibly via inactivating PAR-1 and PAR-4 [25–27], suggesting that this agent may also be effective for atherothrombosis. Rivaroxaban also inhibits inflammatory mediators and promotes plaque stabilization in atherosclerosis animal models [28]. However, there are only few studies focusing on the effects of rivaroxaban on vascular endothelial cells. PAR-1, -2, -3, and -4 are natively expressed in vascular endothelial cells [29]. In an experiment using human umbilical vein endothelial cells, Seki et al. [30] demonstrated that FXa stimulation increased the expression of PAR-1, -2, and -3, but not PAR-4 and that rivaroxaban suppressed the expression of PAR-1 and -2 but not PAR-3. Furthermore, suppression of PAR-2 expression by rivaroxaban was stronger than that of PAR-1 expression. Previously, Fortunato et al. [31] showed that PAR-2 mRNA levels in endothelial colony-forming cells, similar to EPCs, were threefold higher than those in glomerular endothelial cells, whereas that of PAR-1 mRNA was eightfold lower. Therefore, we hypothesized that the enhanced differentiation ability of EPCs by rivaroxaban may be mediated mainly through PAR-2 signaling. As hypothesized, in this study, flow cytometry analysis of PAR-2 siRNA-transfected EPCs showed that PAR-2 knockdown increased the surface expression of VEGFR-1, VEGFR-2, Tie-2, and E-selectin. Moreover, adhesion and tube formation assays of PAR-2 siRNA-transfected EPCs showed that PAR-2 knockdown increased the bioactivities of EPCs. Thus, signaling via PAR-2 suppressed the differentiation of EPCs into endothelial cells, and rivaroxaban enhanced this differentiation possibly in part by inactivating PAR-2. Similarly, Wu et al. [32] observed that rivaroxaban improved neovascularization in the ischemic hindlimb using a streptozotocin-induced diabetic mouse model via enhancement of the migration and senescence of EPCs. The effects of rivaroxaban on neovascularization in ischemic organs may also be advantageous for improving the prognosis of patients with coronary artery disease. In this study, the effects of rivaroxaban on human EPC differentiation ability were assessed using colony-forming assays. Accordingly, the anti-atherogenic effects of rivaroxaban may be in part based on the enhanced differentiation ability of EPCs.

Finally, we evaluated clinically the effects of rivaroxaban on mobilization and differentiation ability of EPCs in 4 patients with coronary artery disease and atrial fibrillation who underwent PCI using drug-eluting stent. In these patients, the anticoagulant was switched from warfarin to rivaroxaban immediately after PCI. Consequently, the number of CD34^+^/KDR^+^cells, which contain abundant amounts of EPCs, increased on day 7 after PCI under the rivaroxaban prescription, compared with baseline under the warfarin prescription, in all patients. In addition, the 2 patients who showed increased numbers of large EPC colonies exhibited better neointimal coverage over the stent struts by OCT observations at 12 months. In our previous study, we observed that the number of circulating bone-marrow-derived EPCs increased after deployment of bare metal stent with a peak at the day 7 but decreased after deployment of drug-eluting stent [14–16]. In addition, we demonstrated that the change in the number of EPCs showed significant positive correlation with the neointimal area measured by OCT observations at chronic phase [16]. Since current clinical observations were performed only in a few patients, we cannot describe anything conclusive. From our results, however, we can envision that rivaroxaban-induced mobilization and differentiation of EPCs may lead to the repair of vascular injury.

### Potential limitations

The current study had several potential limitations. First, we focused on PI3K/Akt/eNOS pathway as an important signaling pathway for differentiation of EPCs. However, we observed phosphorylation of only Akt and eNOS but did not investigate a role the upstream PI3K, e. g., by blocking using its inhibitor Wortmannin. In addition, EPC differentiation is promoted by angiogenetic growth factors including VEGF, as downstream effectors of the eNOS pathway [21]. More comprehensive studies of the signaling pathways mediating EPC differentiation are needed. Second, among 4 PARs, all of which naturally expressed on the endothelial cells, we focused only on PAR-2 as a receptor that associates rivaroxaban to EPC biology. Although suppression of PAR-2 expression by rivaroxaban was stronger than that for PAR-1 [30], we would need to focus also on PAR-1. Finally, the biggest limitation was that the clinical observation was performed only in a few patients. In addition, in these patients, aspirin and warfarin were given until undergoing PCI as baseline antithrombotic therapy, and a P2Y12 inhibitor was added to aspirin simultaneously at switching of warfarin to rivaroxaban immediately after PCI. Therefore, the possibility that the results were affected by the additional P2Y12 inhibitor or dual-antiplatelet therapy (aspirin plus a P2Y12 inhibitor) cannot be denied. Since the effects of switching warfarin to rivaroxaban on mobilization and differentiation ability of the EPCs might be modified by the additional P2Y12 inhibitor or baseline dual-antiplatelet therapy, other groups of patients with antiplatelets therapies alone might be needed as control groups. Taken together, further studies are necessary.

## Conclusions

Rivaroxaban enhanced the differentiation ability of human peripheral blood EPCs and increased the expression of endothelial cell surface markers via Akt and eNOS phosphorylation in human umbilical cord-derived EPCs. These effects of rivaroxaban may be in part based on inactivation of PAR-2. We believe that our findings provided a rationale for the current guidelines that recommend rivaroxaban monotherapy for patients with coronary artery disease complicated with atrial fibrillation 1 year or later after undergoing PCI.

## Supplementary Information

**Additional file 1.**

**Additional file 2.**

**Additional file 3.**

**Additional file 4.**

## Data Availability

Raw data ready to be submitted as needed. R.S. and M.S. can provide the data.
