# Supplementary figures and images for: Effects of the factor Xa inhibitor rivaroxaban on the differentiation of endothelial progenitor cells
Source: BMC Cardiovasc Disord. 2023 Jun 2;23:282. doi: 10.1186/s12872-023-03318-4 (PMC10236699; doi:10.1186/s12872-023-03318-4)

## Slide 1
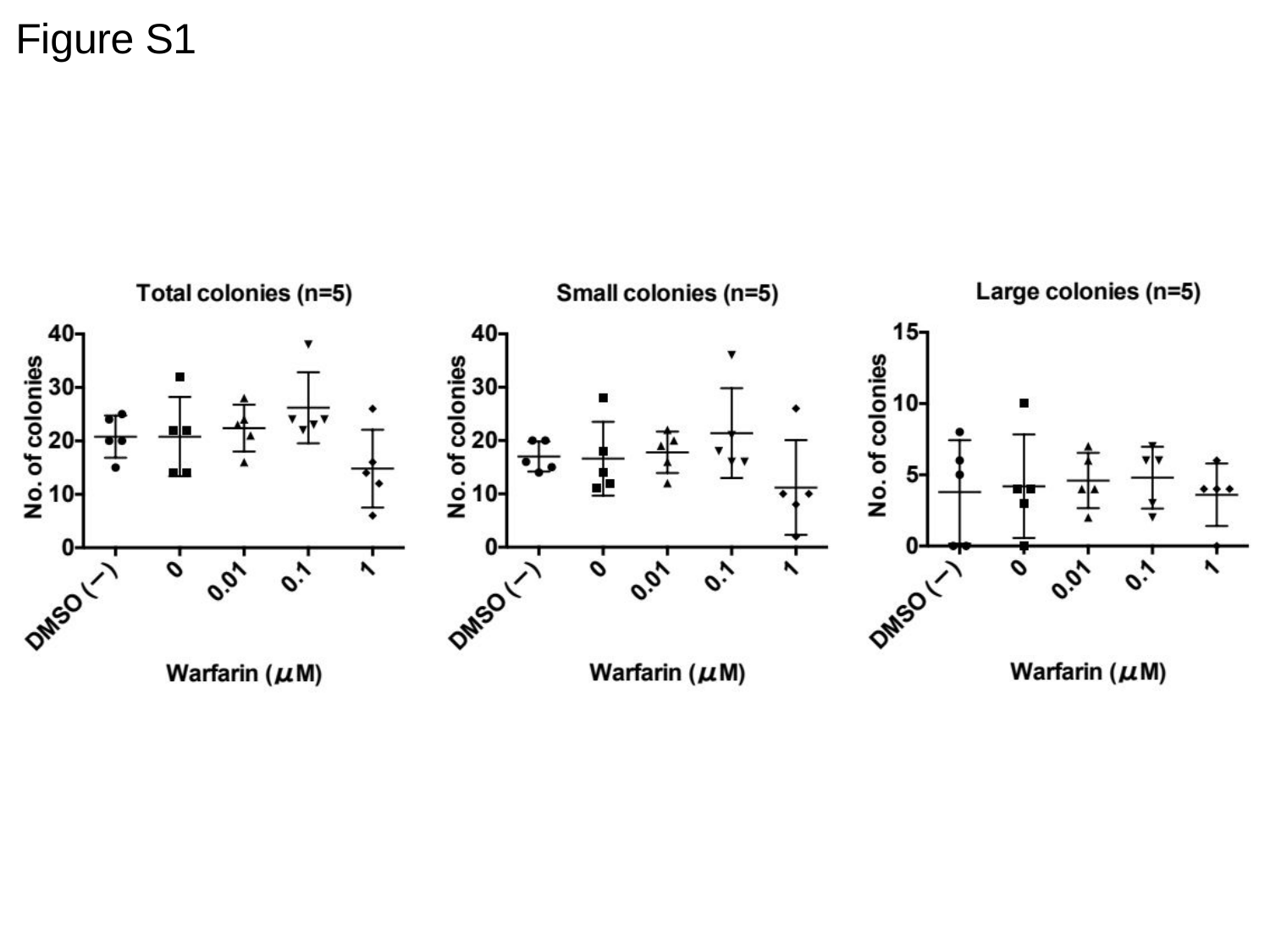

Figure S1

Supplement: Supplementary file 1 — Additional file 1. [file 12872_2023_3318_MOESM1_ESM.pptx]
