# Supplementary material for: Effects of the factor Xa inhibitor rivaroxaban on the differentiation of endothelial progenitor cells
Source: BMC Cardiovasc Disord. 2023 Jun 2;23:282. doi: 10.1186/s12872-023-03318-4 (PMC10236699; doi:10.1186/s12872-023-03318-4)
Supplement: Supplementary file 2 — Additional file 2. [file 12872_2023_3318_MOESM2_ESM.pptx]

## Slide 1
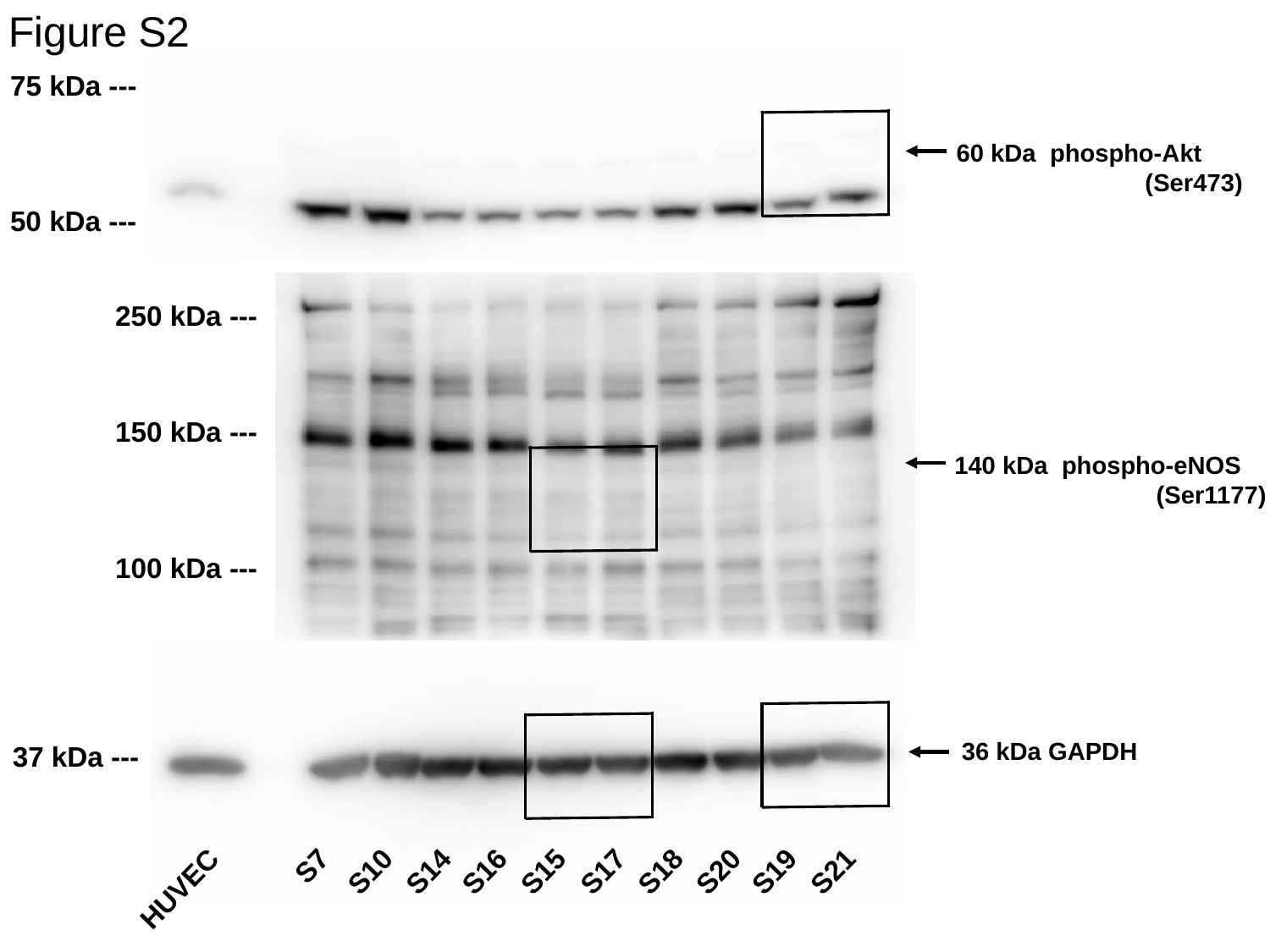

Figure S2
75 kDa ---
60 kDa phospho-Akt
 (Ser473)
50 kDa ---
250 kDa ---
150 kDa ---
140 kDa phospho-eNOS
 　(Ser1177)
100 kDa ---
 36 kDa GAPDH
37 kDa ---
S7
S10
S14
S16
S15
S17
S18
S20
S19
S21
HUVEC
