# Supplementary material for: Effects of the factor Xa inhibitor rivaroxaban on the differentiation of endothelial progenitor cells
Source: BMC Cardiovasc Disord. 2023 Jun 2;23:282. doi: 10.1186/s12872-023-03318-4 (PMC10236699; doi:10.1186/s12872-023-03318-4)
Supplement: Supplementary file 3 — Additional file 3. [file 12872_2023_3318_MOESM3_ESM.pptx]

## Slide 1
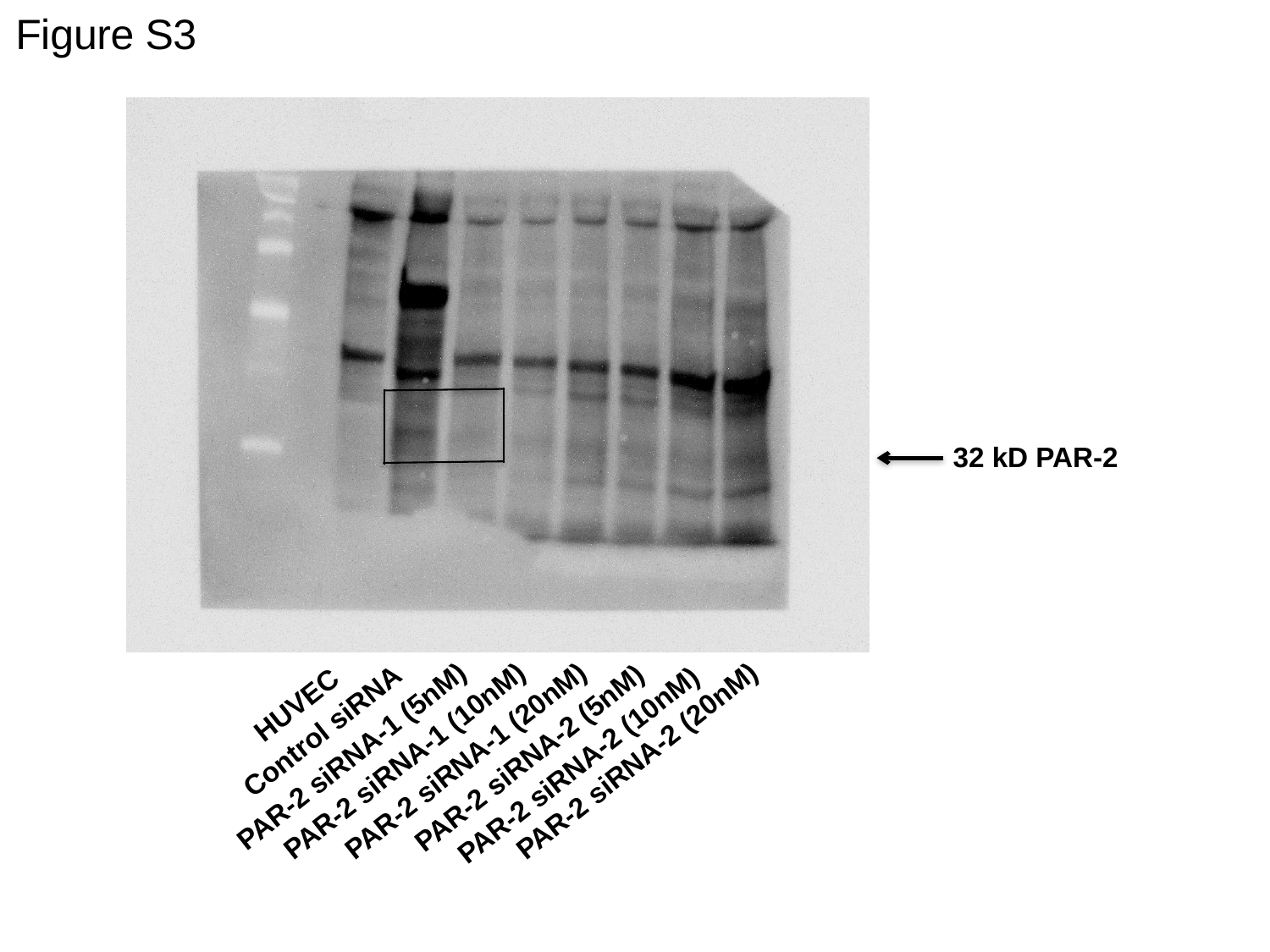

Figure S3
32 kD PAR-2
HUVEC
Control siRNA
PAR-2 siRNA-1 (5nM)
PAR-2 siRNA-2 (5nM)
PAR-2 siRNA-1 (10nM)
PAR-2 siRNA-1 (20nM)
PAR-2 siRNA-2 (20nM)
PAR-2 siRNA-2 (10nM)
